# Physical Activity During Adolescence and Early-adulthood and Ovarian Cancer Among Women with a *BRCA1* or *BRCA2* Mutation

**DOI:** 10.1158/2767-9764.CRC-23-0223

**Published:** 2023-11-28

**Authors:** Emma Guyonnet, Shana J. Kim, Yue Yin Xia, Vasily Giannakeas, Jan Lubinski, Susan Randall Armel, Andrea Eisen, Louise Bordeleau, Charis Eng, Olufunmilayo I. Olopade, Nadine Tung, William D. Foulkes, Fergus J. Couch, Amber M. Aeilts, Steven A. Narod, Joanne Kotsopoulos

**Affiliations:** 1 Women's College Research Institute, Women's College Hospital, Toronto, Ontario, Canada.; 2 Department of Nutritional Sciences, University of Toronto, Toronto, Ontario, Canada.; 3 Dalla Lana School of Public Health, University of Toronto, Toronto, Ontario, Canada.; 4 Department of Molecular Genetics, University of Toronto, Toronto, Ontario, Canada.; 5 ICES, Toronto, Ontario, Canada.; 6 International Hereditary Cancer Center, Department of Genetics and Pathology, Pomeranian Medical University, Szczecin, Poland.; 7 Bhalwani Familial Cancer Clinic, Princess Margaret Cancer Centre, Toronto, Ontario, Canada.; 8 Department of Medical Genetics, University of Toronto, Toronto, Ontario, Canada.; 9 Toronto-Sunnybrook Regional Cancer Center, Toronto, Canada.; 10 Department of Oncology, Juravinski Cancer Centre, Hamilton, Canada.; 11 Genomic Medicine Institute, Center for Personalised Genetic Healthcare, Cleveland Clinic, Cleveland, Ohio.; 12 Department of Medicine and Human Genetics, University of Chicago, Chicago, Illinois.; 13 Beth Israel Deaconess Medical Center, Boston, Massachusetts.; 14 Program in Cancer Genetics, Department of Oncology and Human Genetics, McGill University, Montréal, Quebec, Canada.; 15 Division of Experimental Pathology and Laboratory Medicine, Department of Laboratory Medicine and Pathology, Mayo Clinic, Rochester, Minnesota.; 16 Division of Human Genetics, Ohio State University Medical Center, Comprehensive Cancer Center, Columbus, Ohio.

## Abstract

In the general population, physical activity has been associated with a lower risk of several cancers; however, the evidence for ovarian cancer is not clear. It is suggested that early-life physical activity may differentially impact risk. Whether this is true among women at high risk due to a pathogenic variant (mutation) in the *BRCA1* or *BRCA2* genes has not been evaluated. Thus, we performed a matched case–control study to evaluate the association between adolescent and early-adulthood physical activity and ovarian cancer. *BRCA* mutation carriers who completed a research questionnaire on various exposures and incident disease and with data available on physical activity were eligible for inclusion. Self-reported activity at ages 12–13, 14–17, 18–22, 23–29, and 30–34 was used to calculate the average metabolic equivalent of task (MET)-hours/week for moderate, vigorous, and total physical activity during adolescence (ages 12–17) and early-adulthood (ages 18–34). Conditional logistic regression was used to estimate the OR and 95% confidence intervals (CI) of invasive ovarian cancer associated with physical activity. This study included 215 matched pairs (mean age = 57.3). There was no association between total physical activity during adolescence (OR_high vs. low_ = 0.91; 95% CI: 0.61–1.36; *P*_trend_ = 0.85), early-adulthood (OR_high vs. low_ = 0.78; 95% CI: 0.51–1.20; *P*_trend_ = 0.38) and overall (OR_high vs. low_ = 0.81; 95% CI: 0.54–1.23; *P*_trend_ = 0.56) and ovarian cancer. Findings were similar for moderate (*P*_trend_ ≥ 0.25) and vigorous (*P*_trend_ ≥ 0.57) activity. These findings do not provide evidence for an association between early-life physical activity and *BRCA*-ovarian cancer; however, physical activity should continue to be encouraged to promote overall health.

**Significance:**

In this matched case–control study, we observed no association between physical activity during adolescence or early-adulthood and subsequent risk of ovarian cancer. These findings do not provide evidence for an association between early-life physical activity and *BRCA*-ovarian cancer; however, being active remains important to promote overall health and well-being.

## Introduction

There is an established body of evidence, both epidemiologic and mechanistic, that physical activity is inversely associated with the risk of developing various cancers, although its role in the etiology of ovarian cancer is not clearly defined (1). Case–control studies have generally reported an inverse association between lifetime recreational physical activity and risk of ovarian cancer (2), while more recent prospective studies have either reported no association (2), or in some instances, an increased risk (2). It has been suggested that physical activity during adolescence may play a more important role by delaying menarche and increasing the frequency of anovulatory cycles while physical activity during early-adulthood may do so by altering sex hormone levels (3, 4). Despite this, few studies have examined the association between adolescent and early-adulthood (early-life) physical activity and the risk of this disease (3, 4).

Whether or not early-life physical activity is associated with ovarian cancer in high-risk populations is not known. The lifetime risk of developing ovarian or fallopian tube cancer (ovarian cancer) among women in the general population is 1.7% (5). In contrast, women who carry a pathogenic (or likely pathogenic) variant (mutation hereafter) in the *BRCA1* or *BRCA2* gene face substantially higher risks, estimated at 40% and 20%, respectively (6). Because screening for early detection has not proven to be effective at reducing mortality (7), primary prevention with bilateral salpingo-oophorectomy (oophorectomy) remains the standard of care (8).

Given the highly penetrant nature of these mutations, it is of interest to continue to elucidate the role of nongenetic factors, including lifestyle or other modifiable factors. Apart from oral contraceptive use and breastfeeding, which are significantly and inversely associated with risk, few other exposures have been shown to impact *BRCA*-associated ovarian cancer risk (9, 10). To our knowledge, there are no studies that have evaluated the association between physical activity during early-life and ovarian cancer specifically in this high-risk population. Thus, we conducted a matched case–control study of physical activity during adolescence as well as early-adulthood and ovarian cancer among *BRCA* mutation carriers enrolled in an ongoing longitudinal study of hereditary cancer and who had information available on lifetime levels of physical activity.

## Materials and Methods

### Study Population

Potentially eligible participants were identified from a longitudinal cohort of *BRCA1* or *BRCA2* mutation carriers enrolled from 80 participating centers across 12 countries as previously described in detail (11). Briefly, eligible women were those with a confirmed pathogenic (or likely pathogenic) mutation in the *BRCA1* or *BRCA2* gene and who were between the ages of 18 and 70. All women sought genetic testing due to a personal or family history of breast and/or ovarian cancer. Various techniques were used to detect mutations, but direct DNA sequencing was used to confirm all identified variants. Written informed consent was obtained from all subjects involved in the study. All procedures performed in studies involving human participants were in accordance with the ethical standards of the institutional and/or national research committee and with the 1964 Helsinki Declaration and its later amendments or comparable ethical standards. The study protocol was approved by the Institutional Review Boards of each participating centre.

### Data Collection

All participants completed a baseline questionnaire at the time of study enrollment (between 1995 and 2018) that collected detailed information on relevant exposures and outcomes, including history of cancer, screening, and surgery, as well as select lifestyle factors. Follow-up questionnaires were administered every 2 years to update exposures and diagnosis of cancer and other diseases. All questionnaires were either mailed or emailed to participants to complete at home or administered over the phone by a research assistant or genetic counselor.

For the current study, we reported on a subset of women who were enrolled in the longitudinal study and had completed a validated supplemental questionnaire (i.e., the Nurses’ Health Study II Physical Activity Questionnaire) that collected detailed information on recreational physical activity between ages 12 and 34 (12). This questionnaire was included in the baseline questionnaire for new participants or in the follow-up questionnaires for women already enrolled in the study. This questionnaire was distributed between the years 2014 and 2018.

### Assessment of Physical Activity

The physical activity questionnaire queried the average amount of time (0 to ≥11 hours/week) participants engaged in: (a) moderate recreational physical activity (i.e., hiking, walking for exercise, casual cycling) and (b) vigorous recreational activity (i.e., running, aerobics, lap swimming) during five predefined age periods: (i) grades 7–8 (ages 12–13), (ii) grades 9–12 (ages 14–17), (iii) ages 18–22, (iv) ages 23–29, and (v) ages 30–34. To account for intensity, a metabolic equivalent of task (MET) score was used to calculate physical activity exposure levels. For moderate physical activity, a MET score of 4.5 was assigned (13), then multiplied by the average number of hours of moderate physical activity reported per week to estimate the MET-hours/week score for each age period. For vigorous physical activity, a MET score of 7 was assigned (13), then multiplied by the average number of hours of vigorous physical activity reported per week to estimate the MET-hours/week score. Total recreational physical activity was then calculated by summing the moderate and vigorous MET-hours/week scores for each age period. For each type of physical activity (i.e., moderate, vigorous, total), the MET-hours/week scores during grades 7–8 and 9–12 were summed and averaged to estimate the average activity during adolescence. MET-hours/week scores for each type of physical activity at ages 18–22, 23–29, and 30–34 was summed and averaged to estimate the average early-adulthood activity*.* MET-hours/week scores during the five predefined age periods were then summed and averaged to estimate the average activity overall. See Supplementary Table S1 for a detailed summary of the exposure classification.

### Case and Control Subjects

**FIGURE 1:**
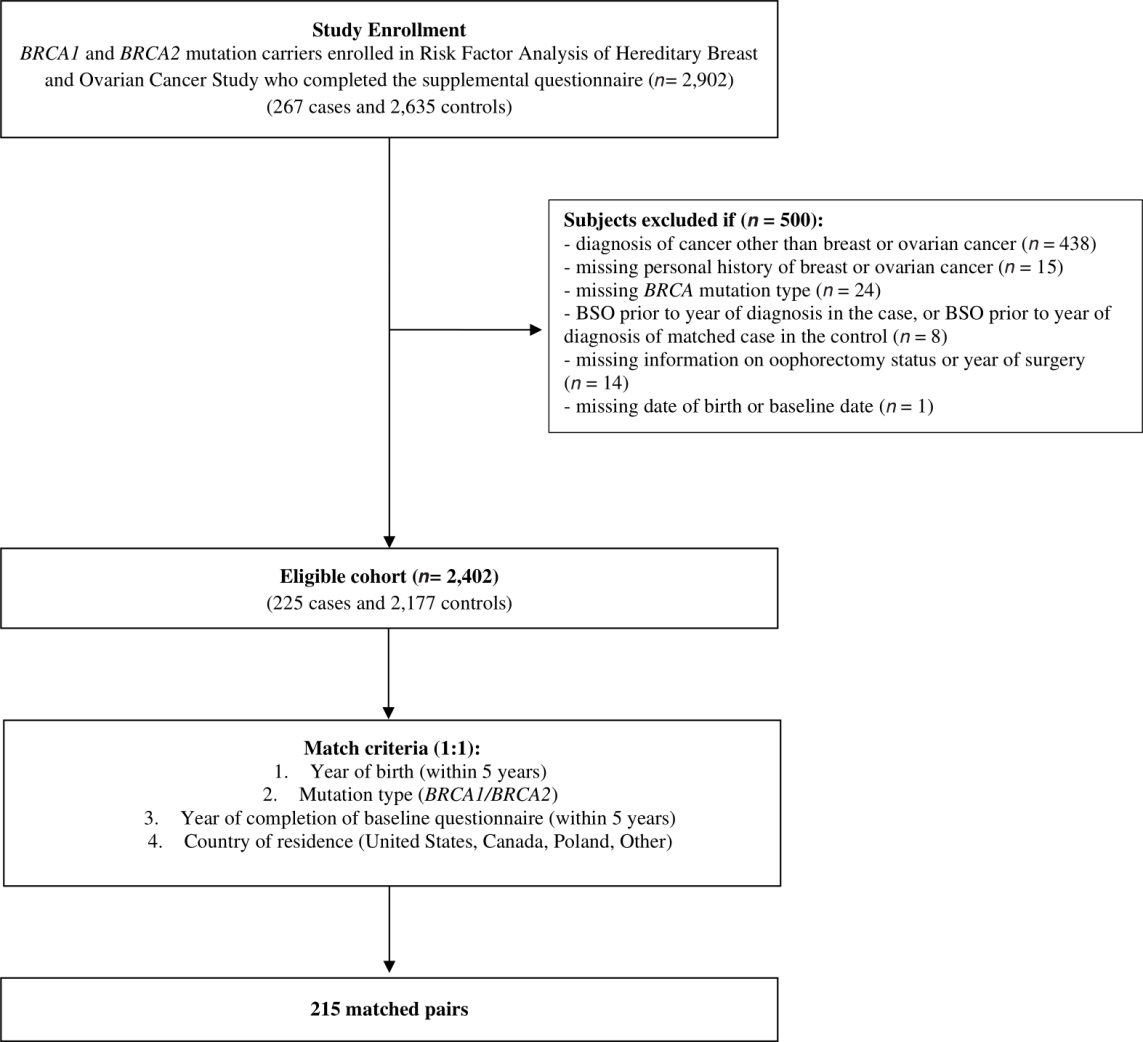
Flow diagram describing subject selection.

There was a total of 2,902 *BRCA1* or *BRCA2* mutation carriers who completed the supplemental questionnaire on physical activity and were potentially eligible for inclusion in the current analysis (Fig. 1). Participants were excluded if they had a previous diagnosis of cancer other than breast or ovarian cancer (*n* = 438), were missing information on personal history of breast or ovarian cancer (*n* = 15), missing *BRCA* mutation type (*n* = 24), had an oophorectomy prior to the date of ovarian cancer diagnosis (*n* = 8), missing information on oophorectomy status or year of surgery (*n* = 14), or missing date of birth or baseline date (*n* = 1). After exclusions, a total of 2,402 subjects were available for analysis, including 225 potential cases and 2,177 potential controls.

Cases were defined as having a self-reported diagnosis of a primary invasive ovarian or fallopian tube cancer. We did not include primary peritoneal cancer in the case definition. For all cases, pathology reports or medical records were requested to obtain information on tumor histology, clinical staging, site of spread, and primary site of origin. Pathology reports were not available for participants diagnosed prior to study enrolment.

Controls were women who never had self-reported ovarian cancer. A control subject was matched to a case only if the date of prophylactic bilateral salpingo-oophorectomy in the matched control occurred at or after the year of ovarian cancer diagnosis of the case. Cases and controls were matched 1:1 by *BRCA* mutation type (*BRCA1* or *BRCA2*), year of birth (±5 years), country of residence (Canada, United States, Poland, Other), and year of completion of the baseline questionnaire (±5 years). In total, 215 matched pairs were identified.

### Statistical Analysis

A matched case–control analysis was employed to evaluate the association between the three types of physical activity (i.e., moderate, vigorous, total) across the three timepoints (i.e., adolescence, early-adulthood, overall) and ovarian cancer. Data for the exposures and covariates were censored at the date of diagnosis of the matched case for the controls and prior to the date of diagnosis of ovarian cancer for the cases.

Physical activity (i.e., moderate, vigorous, total) during each age period (i.e., adolescence, early-adulthood, overall) was categorized as low or high based on the median MET values in the controls. The Student *t* test and *χ*^2^ test were used to compare continuous and categorical subject characteristics between cases and controls, respectively. Conditional logistic regression was used to estimate the OR and 95% confidence intervals (CI) for ovarian cancer associated with moderate, vigorous, and total physical activity during adolescence, early-adulthood, and overall.

Multivariable analysis further adjusted for personal history of breast cancer (no/yes), oral contraceptive use (never/ever), breastfeeding (never/ever), menopausal hormone therapy (MHT) use (never/ever), and tubal ligation (no/yes). Because physical activity may impact upon ovarian cancer risk through its association with body mass index (BMI), an additional model was adjusted for BMI at age 18 (3). Women who had both a *BRCA1* and *BRCA2* mutation were categorized as *BRCA1* mutation carriers due to the higher risk of ovarian cancer associated with *BRCA1* mutations (*n* = 3). Subjects who were missing age at menarche (*n* = 8) or BMI at age 18 (*n* = 34) were assigned the median of the entire sample. Women who were diagnosed with ovarian cancer between the ages of 18 and 34 were excluded from the analyses of physical activity during early-adulthood and overall (*n* = 20). Multivariable model selection was based on the purposeful selection of covariates method described by Hosmer and colleagues (14). The *P* for trend was estimated by modeling each unit increase in physical activity (MET-hours/week).

We evaluated effect modification of total physical activity and ovarian cancer by stratifying our analyses by gene (*BRCA1* or *BRCA2*) and menopausal status (premenopausal or postmenopausal). Controls were categorized as postmenopausal if their age at menopause was earlier than their age at censor date. Cases were classified as postmenopausal if they had stopped menstruating for at least 1 year prior to their date of diagnosis due to reasons other than cancer. In a secondary analysis, we stratified our analyses by BMI at age 18 based on the median BMI at age 18 of the entire cohort (<20.5 kg/m^2^ or ≥20.5 kg/m^2^). The statistical significance of the interaction term was assessed using the likelihood ratio test.

In a supplemental analysis, we further subdivided overall physical activity for the three physical activity exposures (i.e., moderate, vigorous, total) into tertiles based on the distribution in the controls.

All statistical analyses were conducted using SAS OnDemand (SAS Institute). All *P* values were two sided and considered statistically significant if *P* < 0.05.

### Data Availability

Access to data described in the article may be provided through a formal application and review process to the principal investigator. If accepted, sharing may only be approved upon acceptable ethics and contract execution.

## Results

Table 1 summarizes the characteristics of the 430 women included in the final analysis by case or control status. Cases and controls did not differ with respect to year of birth, year of baseline, country of residence, and *BRCA* mutation type after matching. A significantly lower proportion of cases than controls reported a history of oral contraceptive use (24.2% vs. 33.5%; *P* = 0.03). Ovarian cancer cases were older at the onset of menopause than controls (48.8 vs. 47.3 years old; *P* = 0.03). Cases were more likely to have used MHT (15.3% vs. 7.9%; *P* = 0.02) and less likely to have a history of breast cancer than controls (19.5% vs. 31.2%; *P* = 0.006).

**TABLE 1:** Comparison of cases and controls with a *BRCA1* or *BRCA2* mutation

	Cases	Controls	
Characteristic	(*n* = 215)	(*n* = 215)	*P*
Year of birth, mean (range)	1957.4 (1935–1985)	1958.6 (1934–1989)	Matched
Year of baseline, mean (range)	2010.3 (1996–2018)	2010.4 (1995–2018)	Matched
Age at ovarian cancer diagnosis, mean (SD)	50.5 (8.3)	n/a	n/a
Country of residence, *n* (%)			Matched
United States	25 (11.6)	25 (11.6)	
Canada	73 (34.0)	73 (34.0)	
Poland	113 (52.6)	113 (52.6)	
Other	4 (1.8)	4 (1.8)	
Mutation, *n* (%)			Matched
*BRCA1*	173 (80.5)	173 (80.5)	
*BRCA2*	42 (19.5)	42 (19.5)	
Personal history of breast cancer, *n* (%)			0.006
No	173 (80.5)	148 (68.8)	
Yes	42 (19.5)	67 (31.2)	
Breastfeeding history, *n* (%)			0.59
No	63 (29.3)	58 (27.0)	
Yes	152 (70.7)	157 (73.0)	
BMI at age 18 (kg/m^2^), mean (SD)	21.0 (2.9)	20.6 (2.5)	0.11
BMI at age 18 (kg/m^2^), *n* (%)			0.29
<20.5	93 (43.3)	104 (48.4)	
≥20.5	122 (56.7)	112 (51.6)	
Current BMI (kg/m^2^), mean (SD)	26.8 (5.0)	26.7 (5.1)	0.86
Age at menarche (year), mean (SD)	13.0 (1.5)	13.2 (1.5)	0.19
Age at menopause (year), mean (SD)	48.8 (4.8)	47.3 (4.9)	0.03
Menopausal status^a^, *n* (%)			0.77
Premenopausal	110 (51.2)	113 (52.6)	
Postmenopausal	105 (48.8)	102 (47.4)	
Parity, *n* (%)			0.66
Nulliparous	29 (13.5)	26 (12.1)	
Parous	186 (86.5)	189 (87.9)	
Oral contraceptive use, *n* (%)			0.03
Never	163 (75.8)	143 (66.5)	
Ever	52 (24.2)	72 (33.5)	
MHT use, *n* (%)			0.02
Never	182 (84.7)	198 (92.1)	
Ever	33 (15.3)	17 (7.9)	
Tubal ligation, *n* (%)			0.06
Never	189 (87.9)	175 (81.4)	
Ever	26 (12.1)	40 (18.6)	
Hysterectomy, *n* (%)			1.00
Never	206 (95.8)	206 (95.8)	
Ever	9 (4.2)	9 (4.2)	
Alcohol consumption, *n* (%)			0.71
Never	42 (19.5)	39 (18.1)	
Ever	173 (80.5)	176 (81.9)	
Smoking status, *n* (%)			0.85
Never	125 (58.1)	123 (57.2)	
Ever	90 (41.9)	92 (42.8)	
Moderate physical activity (MET-hours/week)			
Adolescent, mean ± SD (range)	15.2 ± 15.3 (0–49.5)	15.2 ± 15.6 (0–49.5)	0.96
Early-adulthood, mean ± SD (range)	15.2 ± 14.8 (0–49.5)	16.8 ± 15.9 (0–49.5)	0.30
Overall, mean ± SD (range)	15.1 ± 14.1 (0–49.5)	16.3 ± 14.9 (0–49.5)	0.43
Vigorous physical activity (MET-hours/week)			
Adolescent, mean ± SD (range)	22.7 ± 24.6 (0–77)	21.7 ± 23.6 (0–77)	0.65
Early-adulthood, mean ± SD (range)	18.4 ± 20.4 (0–77)	19.1 ± 21.2 (0–77)	0.73
Overall, mean ± SD (range)	20.0 ± 20.7 (0–77)	20.3 ± 20.7 (0–77)	0.90
Total physical activity (MET-hours/week)			
Adolescent, mean ± SD (range)	37.9 ± 35.9 (0–126.5)	36.9 ± 35.4 (0–126.5)	0.78
Early-adulthood, mean ± SD (range)	33.7 ± 31.5 (0–126.5)	35.9 ± 33.0 (0–126.5)	0.48
Overall, mean ± SD (range)	35.1 ± 31.6 (0–126.5)	36.5 ± 32.1 (0–126.5)	0.66

Abbreviation: SD, standard deviation.

^a^Controls and cases with age at menopause less than their age at censor date were classified as postmenopausal. Cases with age of menopause equal to their age at diagnosis due to cancer-related surgery were categorized as premenopausal.

The relationship between moderate physical activity and ovarian cancer among *BRCA* mutation carriers is summarized in Table 2. There was no significant association between moderate physical activity during adolescence (≥11.3 vs. <11.3 MET-hours/week, OR = 1.13; 95% CI: 0.75–1.72), early-adulthood (≥12.8 vs. <12.8 MET-hours/week, OR = 0.81; 95% CI: 0.53–1.25) and overall (≥12.2 vs. <12.2 MET-hours/week, OR = 1.00; 95% CI: 0.66–1.51) and the odds of having ovarian cancer (*P*_trend_ ≥ 0.26).

**TABLE 2:** Association between moderate physical activity (in MET-hours/week) and ovarian cancer among women with a *BRCA1* or *BRCA2* mutation

Moderate physical activity (MET-hours/week)	Cases/ controls	Univariate OR (95% CI)	*P*	Multivariable OR (95% CI)^a^	*P*	Multivariable OR (95% CI)^b^	*P*
Adolescent							
<11.3 [0.0 (0–9.0)]^c^	102/107	Ref.	Ref.	Ref.	Ref.	Ref.	Ref.
≥11.3 [24.8 (11.3–49.5)]	113/108	1.11 (0.75–1.64)	0.62	1.13 (0.75–1.72)	0.56	1.14 (0.75–1.73)	0.54
*P*_trend_			0.96		0.84		0.87
Early-adulthood							
<12.8 [0.8 (0.0–11.3)]	101/93	Ref.	Ref.	Ref.	Ref.	Ref.	Ref.
≥12.8 [24.8 (12.8–49.5)]	104/112	0.85 (0.57–1.26)	0.41	0.81 (0.53–1.25)	0.34	0.80 (0.52–1.24)	0.32
*P*_trend_			0.28		0.26		0.25
Overall^d^							
<12.2 [1.4 (0.0–11.7)]	96/97	Ref.	Ref.	Ref.	Ref.	Ref.	Ref.
≥12.2 [23.9 (12.2–49.5)]	109/108	1.02 (0.69–1.51)	0.92	1.00 (0.66–1.51)	1.00	1.00 (0.66–1.52)	1.00
*P*_trend_			0.40		0.36		0.35

Abbreviations: CI, confidence interval; OR, odds ratio.

^a^Adjusted for personal history of breast cancer (no/yes), oral contraceptive use (never/ever), breastfeeding (never/ever), MHT use (never/ever), and tubal ligation (no/yes).

^b^Adjusted for personal history of breast cancer (no/yes), oral contraceptive use (never/ever), breastfeeding (never/ever), MHT use (never/ever), tubal ligation (no/yes), and BMI at age 18.

^c^All such values are [median (range)].

^d^Overall (ages 12–34) was calculated by summing and averaging the metabolic equivalent of the five predefined age periods.

We next evaluated the association between vigorous physical activity across each age period and ovarian cancer (Table 3). There was no association between vigorous physical activity during adolescence (≥17.5 vs. <17.5 MET-hours/week, OR = 0.85; 95% CI: 0.57–1.28), nor early-adulthood (≥11.7 vs. <11.7 MET-hours/week, OR = 0.87; 95% CI: 0.57–1.33) or overall (≥16.1 vs. <16.1 MET-hours/week, OR = 0.76; 95% CI: 0.50–1.15) and the odds of having ovarian cancer (*P*_trend_ ≥ 0.57).

**TABLE 3:** Association between vigorous physical activity (in MET-hours/week) and ovarian cancer among women with a *BRCA1* or *BRCA2* mutation

Vigorous physical activity (MET-hours/week)	Cases/ controls	Univariate OR (95% CI)	*P*	Multivariable OR (95% CI)^a^	*P*	Multivariable OR (95% CI)^b^	*P*
Adolescent							
<17.5 [0.0 (0.0–10.5)]^c^	111/105	Ref.	Ref.	Ref.	Ref.	Ref.	Ref.
≥17.5 [38.5 (17.5–77)]	104/110	0.90 (0.61–1.31)	0.56	0.85 (0.57–1.28)	0.44	0.85 (0.57–1.28)	0.44
*P*_trend_			0.66		0.73		0.71
Early-adulthood							
<11.7 [0.0 (0.0–11.7)]	107/103	Ref.	Ref.	Ref.	Ref.	Ref.	Ref.
≥11.7 [31.5 (12.8–77)]	98/102	0.92 (0.62–1.37)	0.69	0.87 (0.57–1.33)	0.53	0.88 (0.57–1.34)	0.54
*P*_trend_			0.72		0.57		0.62
Overall^d^							
<16.1 [0.0 (0.0–15.4)]	110/97	Ref.	Ref.	Ref.	Ref.	Ref.	Ref.
≥16.1 [32.9 (16.1–77)]	95/108	0.78 (0.54–1.15)	0.21	0.76 (0.50–1.15)	0.20	0.77 (0.51–1.16)	0.20
*P*_trend_			0.90		0.75		0.80

Abbreviations: CI, confidence interval; OR, odds ratio.

^a^Adjusted for personal history of breast cancer (no/yes), oral contraceptive use (never/ever), breastfeeding (never/ever), MHT use (never/ever), and tubal ligation (no/yes).

^b^Adjusted for personal history of breast cancer (no/yes), oral contraceptive use (never/ever), breastfeeding (never/ever), MHT use (never/ever), tubal ligation (no/yes), and BMI at age 18.

^c^All such values are [median (range)].

^d^Overall (ages 12–34) was calculated by summing and averaging the metabolic equivalent of the five predefined age periods.

Table 4 summarizes the findings for the association between total physical activity and ovarian cancer. There was no association between total physical activity during adolescence (≥31.3 vs. <31.3 MET-hours/week, OR = 0.91; 95% CI: 0.61–1.36), early-adulthood (≥32.4 vs. <32.4 MET-hours/week, OR = 0.78; 95% CI: 0.51–1.19) and overall (≥33.3 vs. <33.3 MET-hours/week, OR = 0.81; 95% CI: 0.54–1.22) and the odds of having ovarian cancer (*P*_trend_ ≥ 0.36).

**TABLE 4:** Association between total physical activity (in MET-hours/week) and ovarian cancer among women with a *BRCA1* or *BRCA2* mutation

Total physical activity (MET-hours/week)^a^	Cases/ controls	Univariate OR (95% CI)	*P*	Multivariable OR (95% CI)^b^	*P*	Multivariable OR (95% CI)^c^	*P*
Adolescent							
<31.3 [6.75 (0–29)]^d^	110/105	Ref.	Ref.	Ref.	Ref.	Ref.	Ref.
≥31.3 [58.8 (31.3–126.5)]	105/110	0.91 (0.63–1.33)	0.63	0.91 (0.61–1.36)	0.64	0.91 (0.61–1.36)	0.65
*P*_trend_			0.78		0.89		0.85
Early-adulthood							
<32.4 [6.75 (0.0–32.4)]	114/103	Ref.	Ref.	Ref.	Ref.	Ref.	Ref.
≥32.4 [55.6 (32.6–126.5)]	91/102	0.80 (0.54–1.19)	0.27	0.78 (0.51–1.19)	0.25	0.78 (0.51–1.20)	0.25
*P*_trend_			0.45		0.36		0.38
Overall^e^							
<33.3 [9.4 (0.0–33.2)]	113/102	Ref.	Ref.	Ref.	Ref.	Ref.	Ref.
≥33.3 [56.7 (33.3–126.5)]	92/103	0.81 (0.55–1.19)	0.28	0.81 (0.54–1.22)	0.32	0.81 (0.54–1.23)	0.32
*P*_trend_			0.64		0.53		0.56

Abbreviations: CI, confidence interval; OR, odds ratio.

^a^Total physical activity was calculated as the sum of moderate and vigorous physical activity.

^b^Adjusted for personal history of breast cancer (no/yes), oral contraceptive use (never/ever), breastfeeding (never/ever), MHT use (never/ever), and tubal ligation (no/yes).

^c^Adjusted for personal history of breast cancer (no/yes), oral contraceptive use (never/ever), breastfeeding (never/ever), MHT use (never/ever), tubal ligation (no/yes), and BMI at age 18.

^d^All such values are [median (range)].

^e^Overall (ages 12–34) was calculated by summing and averaging the metabolic equivalent of the five predefined age periods.

Risk estimates for moderate, vigorous, and total physical activity across each age period were similar after further adjustment for BMI at age 18 (Tables 2–4). There was no association between total physical activity across each age period and ovarian cancer in the analysis stratified by *BRCA* mutation (*P*_interaction_ ≥0.30) or menopausal status (*P*_interaction_ ≥ 0.13), although the stratified analyses were based on small strata (Supplementary Table S2).

In a secondary analysis stratified by BMI at age 18, there was no association between moderate, vigorous, and total physical activity across each age period and ovarian cancer (*P*_interaction_ ≥ 0.17; Supplementary Tables S3–S5).

In the supplemental analysis, there was no association between the highest versus lowest tertile of moderate (*P* ≥ 0.33), vigorous (*P* ≥ 0.39), and total (*P* ≥ 0.79) physical activity overall and ovarian cancer (Supplementary Table S6).

## Discussion

In this matched analysis of 430 women at a high risk of having ovarian cancer due to an inherited *BRCA1* or *BRCA2* mutation, we evaluated whether physical activity during adolescence and early-adulthood was associated with ovarian cancer. Overall, we observed no significant association between the three types of physical activity (i.e., moderate, vigorous, and total) across each age period (i.e., adolescence, early-adulthood, and overall) and ovarian cancer. Although limited by small strata, findings were similar in our analyses stratified by BMI at age 18, *BRCA* gene and menopausal status. To our knowledge, this represents the first analysis of early-life physical activity and ovarian cancer specifically among *BRCA* mutation carriers.

Moderate and vigorous physical activity during adolescence and early-adulthood were not associated with ovarian cancer risk. Studies evaluating this association in noncarriers have been inconclusive with some groups similarly showing no association and others reporting an increased risk (3–4, 15–20). Among 485 ovarian cancer cases, Grundy and colleagues reported no association between moderate to vigorous physical activity at ages 15–19 and 20–44 and ovarian cancer risk (≥78.3 vs. <19.9, MET-hours/week, OR 1.21; 95% CI: 0.88, 1.66 and ≥34.5 vs. <6.1, MET-hours/week, OR 1.20; 95% CI: 0.87, 1.65, respectively; ref. 15). In contrast, in a report from the Nurses’ Health Study (NHS) with 815 incident cases over a follow-up of 24 years, Huang and colleagues showed that premenopausal moderate and vigorous physical activity were associated with an increased risk of ovarian cancer (≥4 vs. 0.25–<1 hour/week of vigorous exercise, HR 1.44; 95% CI: 1.04–1.98; ref. 4). The conflicting findings could be attributed to the misclassification of physical activity (including the definition of physical activity; MET-hours/week vs. hours/week), differing study designs (i.e., case–control vs. prospective) and research methodology.

We also did not observe a significant association between total physical activity (i.e., the sum of moderate and vigorous activity) during adolescence and early-adulthood and ovarian cancer. Studies conducted among women in the general population have also been inconsistent for this exposure. Notably, a recent prospective study from the NHS (including 227 incident cases) that used the same validated physical activity questionnaire as our study reported no association between total physical activity at ages 12–13, 14–17, 18–22, or 12–22 and risk (≥78 vs. <24 MET-hours/week, HRs = 1.34, 1.21, 1.08, and 1.24, respectively; ref. 3). Findings were similar in stratified analyses by early-life BMI (3). Older case–control studies reported similar results (21, 22). In contrast, Huang and colleagues found an increased risk of ovarian cancer among women who engaged in high levels of physical activity prior to menopause (≥27 vs. 3–<9 MET-hours/week, HR = 1.50; 95% CI: 1.13–1.97; ref. 4) while two case–control studies reported an inverse association between the highest level of physical activity (in hours/week) during early-adulthood and ovarian cancer risk (23, 24). The small number of ovarian cancer cases and differences in the ages assessed may explain the discrepant findings. There is also potential for detection bias because women who are more active tend to have a healthier lifestyle and seek medical advice sooner, suggesting that some significant associations observed across studies may be due to noncausal factors (2).

While we did not observe any statistically significant associations between early-life physical activity and ovarian cancer, given our small sample size, we cannot exclude an association, especially as the suggestive inverse associations between physical activity and *BRCA*-ovarian cancer are in line with the levels of cancer risk reduction (relative risk reduction of 8%–25%) from studies of women at baseline population risk (25–26). Similarly, it has been suggested that adolescent and early-adulthood physical activity may also reduce the risk of breast cancer specifically among *BRCA* mutation carriers, further supporting a protective rather than harmful role of exercise for high-risk women (27). There is a growing body of evidence to suggest that both prediagnostic and postdiagnostic recreational physical activity are associated with improved outcomes across several cancer subtypes (28–29). Although the association between physical activity and ovarian cancer risk remains inconclusive, physical activity should continue to be encouraged given its emerging and well-established health benefits across all populations (25–26, 30).

Multiple mechanisms have been proposed through which physical activity may be implicated in the pathogenesis of ovarian cancer including inhibition of ovulation, alterations in circulating sex hormones and insulin-like growth factor 1 (IGF-1), and changes in BMI (31). The effect of early-life physical activity on the development of *BRCA*-associated ovarian cancer is not clear. The incessant ovulation theory suggests that repeated rupture and repair of the ovarian follicle stimulates proliferation and predisposes epithelial cells to carcinogenesis (32). On the basis of this theory, vigorous physical activity in adolescence may decrease cancer risk by delaying menarche and increasing the frequency of anovulatory cycles (33).

Physical activity may cause many hormonal changes (31). Widschwendter and colleagues reported higher estrogen levels among *BRCA* mutation carriers compared with noncarriers, which may play a role in ovarian carcinogenesis in this high-risk population (34). In a meta-analysis of 23 randomized controlled trials, physical activity was associated with a significant decrease in both total estradiol and free estradiol, thereby decreasing cancer risk (35). However, through a negative feedback mechanism, low circulating estrogen levels increase pituitary gonadotropin secretion (32). This hypothesis proposes that high circulating levels of gonadotropins then increase estrogen levels and stimulate ovarian surface proliferation and malignant transformation (32).

This study had several strengths, including a strict matching criterion, the collection of detailed information on various exposures and potential confounders and a standardized questionnaire for physical activity assessment. However, our study had limitations. Only a subset of women from the larger longitudinal study was invited to complete the supplemental physical activity questionnaire, which may have introduced selection bias and affected our sample size, limiting our statistical power. Despite this, this analysis included participants from numerous participating centers allowing for generalizability across various populations. There were a few differences between the cases included in the current analysis and cases in the entire cohort; however, by conducting a matched analysis, we were able to account for key differences and thus, the risk of bias was low (Supplementary Table S7). Survivorship bias is also possible in our study because we also included prevalent cases who were still alive at the time of physical activity assessment. Given the retrospective nature of this study, data collection from women following an ovarian cancer diagnosis may have introduced recall bias in some cases. Use of self-reported physical activity may also have resulted in nondifferential misclassification; however, a validation study of this specific questionnaire showed high reproducibility of recalled activities in early-life (*r* = 0.64; ref. 36). Our analyses stratified by BMI at age 18 were also limited because of insufficient variability in BMI at age 18 levels, which could have impacted our ability to detect a significant modifiable effect across BMI strata. Finally, although we included self-reported ovarian cancer diagnoses, more than 70% of the cases were confirmed through review of pathology reports and medical records.

In summary, these findings do not support a strong association between early-life physical activity and ovarian cancer among women with a *BRCA1* or *BRCA2* mutation. Despite these null findings, women should be encouraged to be active given the well-established role of obesity and physical activity in the prevention of other chronic diseases and for overall health (29, 37).

## Supplementary Material

Supplementary Table 1 Supplementary Table 1 shows a summary of the steps to create exposure variables.

Supplementary Table 2 Supplementary Table 2 shows the association between total physical activity (in MET-hr/week) and ovarian cancer among women with a BRCA1 or BRCA2 mutation, stratified by BRCA mutation type and menopausal status.

Supplementary Table 3 Supplementary Table 3 shows the association between moderate physical activity (in MET-hr/week) and ovarian cancer among women with a BRCA1 or BRCA2 mutation, stratified by BMI at age 18.

Supplementary Table 4 Supplementary Table S4 shows the association between vigorous physical activity (in MET-hr/week) and ovarian cancer among women with a BRCA1 or BRCA2 mutation, stratified by BMI at age 18.

Supplementary Table 5 Supplementary Table 5 shows the association between total physical activity (in MET-hr/week) and ovarian cancer among women with a BRCA1 or BRCA2 mutation, stratified by BMI at age 18.

Supplementary Table 6 Supplementary Table 6 shows the association between physical activity overall (in MET-hr/week) and ovarian cancer among women with a BRCA1 or BRCA2 mutation.

Supplementary Table 7 Supplementary Table 7 shows the comparison of cases in the matched analysis and larger longitudinal study.
