# Supplementary material for: Physical Activity During Adolescence and Early-adulthood and Ovarian Cancer Among Women with a BRCA1 or BRCA2 Mutation
Source: Cancer Res Commun. 2023 Nov 28;3(11):2420–9. doi: 10.1158/2767-9764.CRC-23-0223 (PMC10683556; doi:10.1158/2767-9764.CRC-23-0223)
Supplement: Supplementary Table 1 — shows a summary of the steps to create exposure variables. [file crc-23-0223-s01.docx]

**Supplementary Table S1: Summary of steps to create exposure variables.**

| **Exposures** | **1. Metabolic equivalent of Task (MET)-hour/week for each age group** | **2. Average metabolic equivalent (MET)-hour/week during adolescence, early-adulthood and overall** |
| --- | --- | --- |
| ***Moderate* physical activity** | 1) MET-hr/wk for grades 7-8 = Average number of hours/week*4.5  2) MET-hr/wk for grades 9-12 = Average number of hours/week*4.5  3) MET-hr/wk at ages 18-22 = Average number of hours/week*4.5  4) MET-hr/wk at ages 23-29 = Average number of hours/week*4.5  5) MET-hr/wk at ages 30-34 = Average number of hours/week*4.5 | **Adolescent** = (MET-hr/wk for grades 7-8 + MET-hr/wk for grades 9-12)/2  **Early-adulthood** = (MET-hr/wk at ages 18-22 + MET-hr/wk at ages 22-29 + MET-hr/wk at ages 29-34)/3  **Overall** = (MET-hr/wk for grades 7-8 + MET-hr/wk for grades 9-12 + MET-hr/wk at ages 18-22 + MET-hr/wk at ages 22-29 + MET-hr/wk at ages 29-34)/5 |
| ***Vigorous* physical activity** | 1) MET-hr/wk for grades 7-8 = Average number of hours/week*7  2) MET-hr/wk for grades 9-12 = Average number of hours/week*7  3) MET-hr/wk at ages 18-22 = Average number of hours/week*7  4) MET-hr/wk at ages 23-29 = Average number of hours/week*7  5) MET-hr/wk at ages 30-34 = Average number of hours/week*7 | **Adolescent** = (MET-hr/wk for grades 7-8 + MET-hr/wk for grades 9-12)/2  **Early-adulthood** = (MET-hr/wk at ages 18-22 + MET-hr/wk at ages 22-29 + MET-hr/wk at ages 29-34)/3  **Overall** = (MET-hr/wk for grades 7-8 + MET-hr/wk for grades 9-12 + MET-hr/wk at ages 18-22 + MET-hr/wk at ages 22-29 + MET-hr/wk at ages 29-34)/5 |
| ***Total* physical activity (sum of moderate and vigorous activity)** | 1) Sum of moderate and vigorous MET-hr/wk for grades 7-8  2) Sum of moderate and vigorous MET-hr/wk for grades 9-12  3) Sum of moderate and vigorous MET-hr/wk at ages 18-22  4) Sum of moderate and vigorous MET-hr/wk at ages 23-29  5) Sum of moderate and vigorous MET-hr/wk at ages 30-34 | **Adolescent** = (Total MET-hr/wk for grades 7-8 + Total MET-hr/wk for grades 9-12)/2  **Early-adulthood** = (Total MET-hr/wk at ages 18-22 + Total MET-hr/wk at ages 22-29 + Total MET-hr/wk at ages 29-34)/3  **Overall** = (Total MET-hr/wk for grades 7-8 + Total MET-hr/wk for grades 9-12 + Total MET-hr/wk at ages 18-22 + Total MET-hr/wk at ages 22-29 + Total MET-hr/wk at ages 29-34)/5 |
