# Supplementary material for: Physical Activity During Adolescence and Early-adulthood and Ovarian Cancer Among Women with a BRCA1 or BRCA2 Mutation
Source: Cancer Res Commun. 2023 Nov 28;3(11):2420–9. doi: 10.1158/2767-9764.CRC-23-0223 (PMC10683556; doi:10.1158/2767-9764.CRC-23-0223)
Supplement: Supplementary Table 2 — shows the association between total physical activity (in MET-hr/week) and ovarian cancer among women with a BRCA1 or BRCA2 mutation, stratified by BRCA mutation type and menopausal status. [file crc-23-0223-s02.docx]

**Supplementary Table S2: Association between *total* physical activity (in MET-hr/week) and ovarian cancer among women with a *BRCA1* or *BRCA2* mutation, stratified by *BRCA* mutation type and menopausal status.**

| **Total physical activity (MET-hr/week)^a^** | **Cases/**  **controls** | **Univariate OR (95% CI)** | ***P*** | **Multivariate OR (95% CI)^b^** | ***P*** |
| --- | --- | --- | --- | --- | --- |
| ***BRCA1*** |  |  |  |  |  |
| **Adolescent** |  |  |  |  |  |
| < 31.3 | 89/81 | Ref. | Ref. | Ref. | Ref. |
| ≥ 31.3 | 84/92 | 0.83 (0.55, 1.27) | 0.39 | 0.85 (0.54, 1.35) | 0.50 |
| *P*-trend |  |  | 0.90 |  | 0.76 |
| **Early-adulthood** |  |  |  |  |  |
| < 32.4 | 89/82 | Ref. | Ref. | Ref. | Ref. |
| ≥ 32.4 | 75/82 | 0.84 (0.54, 1.30) | 0.43 | 0.82 (0.50, 1.34) | 0.43 |
| *P*-trend |  |  | 0.51 |  | 0.44 |
| **Overall^c^** |  |  |  |  |  |
| < 33.3 | 91/81 | Ref. | Ref. | Ref. | Ref. |
| ≥ 33.3 | 73/83 | 0.78 (0.51, 1.21) | 0.27 | 0.84 (0.52, 1.36) | 0.48 |
| *P*-trend |  |  | 0.57 |  | 0.46 |
| ***BRCA2*** |  |  |  |  |  |
| **Adolescent** |  |  |  |  |  |
| < 31.3 | 21/24 | Ref. | Ref. | Ref. | Ref. |
| ≥ 31.3 | 21/18 | 1.33 (0.56, 3.16) | 0.51 | 1.55 (0.58, 4.17) | 0.39 |
| *P*-trend |  |  | 0.30 |  | 0.18 |
| **Early-adulthood** |  |  |  |  |  |
| < 32.4 | 25/21 | Ref. | Ref. | Ref. | Ref. |
| ≥ 32.4 | 16/20 | 0.67 (0.27, 1.63) | 0.37 | 0.79 (0.29, 2.19) | 0.65 |
| *P*-trend |  |  | 0.70 |  | 0.97 |
| **Overall^c^** |  |  |  |  |  |
| < 33.3 | 22/21 | Ref. | Ref. | Ref. | Ref. |
| ≥ 33.3 | 19/20 | 0.92 (0.40, 2.08) | 0.83 | 1.06 (0.41, 2.72) | 0.91 |
| *P*-trend |  |  | 0.89 |  | 0.60 |
| **Premenopausal** |  |  |  |  |  |
| **Adolescent** |  |  |  |  |  |
| < 31.3 | 49/52 | Ref. | Ref. | Ref. | Ref. |
| ≥ 31.3 | 61/61 | 0.96 (0.53, 1.74) | 0.88 | 1.14 (0.61, 2.16) | 0.68 |
| *P*-trend |  |  | 0.81 |  | 0.62 |
| **Early-adulthood** |  |  |  |  |  |
| < 32.4 | 54/47 | Ref. | Ref. | Ref. | Ref. |
| ≥ 32.4 | 46/56 | 0.60 (0.29, 1.23) | 0.16 | 0.62 (0.29, 1.31) | 0.21 |
| *P*-trend |  |  | 0.56 |  | 0.74 |
| **Overall^c^** |  |  |  |  |  |
| < 33.3 | 51/47 | Ref. | Ref. | Ref. | Ref. |
| ≥ 33.3 | 49/56 | 0.65 (0.34, 1.25) | 0.20 | 0.70 (0.36, 1.40) | 0.31 |
| *P*-trend |  |  | 0.65 |  | 0.86 |
| **Postmenopausal** |  |  |  |  |  |
| **Adolescent** |  |  |  |  |  |
| < 31.3 | 61/53 | Ref. | Ref. | Ref. | Ref. |
| ≥ 31.3 | 44/49 | 0.91 (0.50, 1.67) | 0.76 | 0.77 (0.39, 1.51) | 0.44 |
| *P*-trend |  |  | 0.83 |  | 0.65 |
| **Early-adulthood** |  |  |  |  |  |
| < 32.4 | 60/56 | Ref. | Ref. | Ref. | Ref. |
| ≥ 32.4 | 45/46 | 0.86 (0.46, 1.61) | 0.63 | 0.75 (0.37, 1.52) | 0.42 |
| *P*-trend |  |  | 0.65 |  | 0.30 |
| **Overall^c^** |  |  |  |  |  |
| < 33.3 | 62/55 | Ref. | Ref. | Ref. | Ref. |
| ≥ 33.3 | 43/47 | 0.78 (0.42, 1.45) | 0.44 | 0.66 (0.33, 1.33) | 0.24 |
| *P*-trend |  |  | 0.86 |  | 0.40 |

Abbreviations: OR, odds ratio; CI, confidence interval.

^a^Total physical activity was calculated as the sum of moderate and vigorous physical activity.

^b^Adjusted for personal history of breast cancer (no/yes), oral contraceptive use (never/ever), breastfeeding (never/ever), HRT use (never/ever) and tubal ligation (no/yes).

^c^Overall (ages 12–34) was calculated by summing and averaging the metabolic equivalent of the five predefined age periods.
