# Supplementary material for: Physical Activity During Adolescence and Early-adulthood and Ovarian Cancer Among Women with a BRCA1 or BRCA2 Mutation
Source: Cancer Res Commun. 2023 Nov 28;3(11):2420–9. doi: 10.1158/2767-9764.CRC-23-0223 (PMC10683556; doi:10.1158/2767-9764.CRC-23-0223)
Supplement: Supplementary Table 3 — shows the association between moderate physical activity (in MET-hr/week) and ovarian cancer among women with a BRCA1 or BRCA2 mutation, stratified by BMI at age 18. [file crc-23-0223-s03.docx]

**Supplementary Table S3: Association between *moderate* physical activity (in MET-hr/week) and ovarian cancer among women with a *BRCA1* or *BRCA2* mutation, stratified by BMI at age 18.**

| **Moderate physical activity (MET-hr/week)** | **Cases/**  **controls** | **Univariate OR (95% CI)** | ***P*** | **Multivariable OR (95% CI)^a^** | ***P*** |
| --- | --- | --- | --- | --- | --- |
| **BMI at age 18 < 20.5 kg/m^2^** |  |  |  |  |  |
| **Adolescent** |  |  |  |  |  |
| < 11.3 | 45/52 | Ref. | Ref. | Ref. | Ref. |
| ≥ 11.3 | 48/52 | 0.82 (0.34, 1.97) | 0.66 | 0.75 (0.23, 2.45) | 0.63 |
| *P*-trend |  |  | 0.26 |  | 0.44 |
| **Early-adulthood** |  |  |  |  |  |
| < 12.8 | 44/46 | Ref. | Ref. | Ref. | Ref. |
| ≥ 12.8 | 44/54 | 0.91 (0.39, 2.14) | 0.83 | 0.78 (0.23, 2.62) | 0.68 |
| *P*-trend |  |  | 0.32 |  | 0.57 |
| **Overall^b^** |  |  |  |  |  |
| < 12.2 | 41/48 | Ref. | Ref. | Ref. | Ref. |
| ≥ 12.2 | 47/52 | 1.08 (0.49, 2.37) | 0.84 | 1.01 (0.33, 3.06) | 0.99 |
| *P*-trend |  |  | 0.27 |  | 0.48 |
| **BMI at age 18 ≥ 20.5 kg/m^2^** |  |  |  |  |  |
| **Adolescent** |  |  |  |  |  |
| < 11.3 | 57/55 | Ref. | Ref. | Ref. | Ref. |
| ≥ 11.3 | 65/56 | 1.31 (0.64, 2.69) | 0.47 | 1.16 (0.53, 2.53) | 0.71 |
| *P*-trend |  |  | 0.95 |  | 0.43 |
| **Early-adulthood** |  |  |  |  |  |
| < 12.8 | 57/47 | Ref. | Ref. | Ref. | Ref. |
| ≥ 12.8 | 60/58 | 0.88 (0.44, 1.77) | 0.72 | 0.85 (0.39, 1.82) | 0.67 |
| *P*-trend |  |  | 0.72 |  | 0.46 |
| **Overall^b^** |  |  |  |  |  |
| < 12.2 | 55/49 | Ref. | Ref. | Ref. | Ref. |
| ≥ 12.2 | 62/56 | 1.00 (0.50, 2.00) | 1.00 | 0.93 (0.43, 1.98) | 0.85 |
| *P*-trend |  |  | 0.74 |  | 0.38 |

Abbreviations: OR, odds ratio; CI, confidence interval.

^a^Adjusted for personal history of breast cancer (no/yes), oral contraceptive use (never/ever), breastfeeding (never/ever), HRT use (never/ever) and tubal ligation (no/yes).

^b^Overall (ages 12–34) was calculated by summing and averaging the metabolic equivalent of the five predefined age periods.
