# Supplementary material for: Physical Activity During Adolescence and Early-adulthood and Ovarian Cancer Among Women with a BRCA1 or BRCA2 Mutation
Source: Cancer Res Commun. 2023 Nov 28;3(11):2420–9. doi: 10.1158/2767-9764.CRC-23-0223 (PMC10683556; doi:10.1158/2767-9764.CRC-23-0223)
Supplement: Supplementary Table 4 — Supplementary Table S4 shows the association between vigorous physical activity (in MET-hr/week) and ovarian cancer among women with a BRCA1 or BRCA2 mutation, stratified by BMI at age 18. [file crc-23-0223-s04.docx]

**Supplementary Table S4: Association between *vigorous* physical activity (in MET-hr/week) and ovarian cancer among women with a *BRCA1* or *BRCA2* mutation, stratified by BMI at age 18.**

| **Vigorous physical activity (MET-hr/week)** | **Cases/**  **controls** | **Univariate OR (95% CI)** | ***P*** | **Multivariable OR (95% CI)^a^** | ***P*** |
| --- | --- | --- | --- | --- | --- |
| **BMI at age 18 < 20.5 kg/m^2^** |  |  |  |  |  |
| **Adolescent** |  |  |  |  |  |
| < 17.5 | 46/51 | Ref. | Ref. | Ref. | Ref. |
| ≥ 17.5 | 47/53 | 1.83 (0.68, 4.96) | 0.23 | 4.12 (0.85, 19.9) | 0.08 |
| *P*-trend |  |  | 0.17 |  | 0.16 |
| **Early-adulthood** |  |  |  |  |  |
| < 11.7 | 47/52 | Ref. | Ref. | Ref. | Ref. |
| ≥ 11.7 | 41/48 | 1.33 (0.56, 3.16) | 0.51 | 1.97 (0.52, 7.48) | 0.32 |
| *P*-trend |  |  | 0.27 |  | 0.44 |
| **Overall^b^** |  |  |  |  |  |
| < 16.1 | 48/48 | Ref. | Ref. | Ref. | Ref. |
| ≥ 16.1 | 40/52 | 1.38 (0.55, 3.42) | 0.49 | 3.01 (0.61, 14.8) | 0.17 |
| *P*-trend |  |  | 0.23 |  | 0.31 |
| **BMI at age 18 ≥ 20.5 kg/m^2^** |  |  |  |  |  |
| **Adolescent** |  |  |  |  |  |
| < 17.5 | 65/54 | Ref. | Ref. | Ref. | Ref. |
| ≥ 17.5 | 57/57 | 1.06 (0.55, 2.05) | 0.87 | 0.92 (0.45, 1.90) | 0.83 |
| *P*-trend |  |  | 0.48 |  | 0.76 |
| **Early-adulthood** |  |  |  |  |  |
| < 11.7 | 60/51 | Ref. | Ref. | Ref. | Ref. |
| ≥ 11.7 | 57/54 | 1.31 (0.64, 2.69) | 0.47 | 1.11 (0.49, 2.50) | 0.81 |
| *P*-trend |  |  | 0.51 |  | 0.83 |
| **Overall^b^** |  |  |  |  |  |
| < 16.1 | 62/49 | Ref. | Ref. | Ref. | Ref. |
| ≥ 16.1 | 55/56 | 0.94 (0.49, 1.83) | 0.87 | 0.82 (0.39, 1.70) | 0.59 |
| *P*-trend |  |  | 0.49 |  | 0.81 |

Abbreviations: OR, odds ratio; CI, confidence interval.

^a^Adjusted for personal history of breast cancer (no/yes), oral contraceptive use (never/ever), breastfeeding (never/ever), HRT use (never/ever) and tubal ligation (no/yes).

^b^Overall (ages 12–34) was calculated by summing and averaging the metabolic equivalent of the five predefined age periods.
