# Supplementary material for: Physical Activity During Adolescence and Early-adulthood and Ovarian Cancer Among Women with a BRCA1 or BRCA2 Mutation
Source: Cancer Res Commun. 2023 Nov 28;3(11):2420–9. doi: 10.1158/2767-9764.CRC-23-0223 (PMC10683556; doi:10.1158/2767-9764.CRC-23-0223)
Supplement: Supplementary Table 5 — shows the association between total physical activity (in MET-hr/week) and ovarian cancer among women with a BRCA1 or BRCA2 mutation, stratified by BMI at age 18. [file crc-23-0223-s05.docx]

**Supplementary Table S5: Association between *total* physical activity (in MET-hr/week) and ovarian cancer among women with a *BRCA1* or *BRCA2* mutation, stratified by BMI at age 18.**

| **Total physical activity**  **(MET-hr/week) ^b^** | **Cases/**  **controls** | **Univariate OR (95% CI)** | ***P*** | **Multivariable OR (95% CI)^a^** | ***P*** |
| --- | --- | --- | --- | --- | --- |
| **BMI at age 18 < 20.5 kg/m^2^** |  |  |  |  |  |
| **Adolescent** |  |  |  |  |  |
| < 31.3 | 46/53 | Ref. | Ref. | Ref. | Ref. |
| ≥ 31.3 | 47/51 | 1.71 (0.68, 4.35) | 0.26 | 2.69 (0.79, 9.20) | 0.12 |
| *P*-trend |  |  | 0.16 |  | 0.19 |
| **Early-adulthood** |  |  |  |  |  |
| < 32.4 | 50/51 | Ref. | Ref. | Ref. | Ref. |
| ≥ 32.4 | 38/49 | 1.20 (0.52, 2.78) | 0.67 | 1.58 (0.44, 5.70) | 0.49 |
| *P*-trend |  |  | 0.24 |  | 0.42 |
| **Overall^c^** |  |  |  |  |  |
| < 33.3 | 48/53 | Ref. | Ref. | Ref. | Ref. |
| ≥ 33.3 | 40/47 | 1.44 (0.62, 3.38) | 0.40 | 1.93 (0.52, 7.12) | 0.32 |
| *P*-trend |  |  | 0.20 |  | 0.32 |
| **BMI at age 18 ≥ 20.5 kg/m^2^** |  |  |  |  |  |
| **Adolescent** |  |  |  |  |  |
| < 31.3 | 64/52 | Ref. | Ref. | Ref. | Ref. |
| ≥ 31.3 | 58/59 | 1.00 (0.54, 1.86) | 1.00 | 0.88 (0.44, 1.74) | 0.71 |
| *P*-trend |  |  | 0.64 |  | 0.88 |
| **Early-adulthood** |  |  |  |  |  |
| < 32.4 | 64/52 | Ref. | Ref. | Ref. | Ref. |
| ≥ 32.4 | 53/53 | 1.13 (0.57, 2.27) | 0.72 | 0.91 (0.41, 2.01) | 0.82 |
| *P*-trend |  |  | 0.83 |  | 0.78 |
| **Overall^c^** |  |  |  |  |  |
| < 33.3 | 65/49 | Ref. | Ref. | Ref. | Ref. |
| ≥ 33.3 | 52/56 | 1.06 (0.55, 2.05) | 0.87 | 0.95 (0.46, 1.97) | 0.89 |
| *P*-trend |  |  | 0.78 |  | 0.76 |

Abbreviations: OR, odds ratio; CI, confidence interval.

^a^Adjusted for personal history of breast cancer (no/yes), oral contraceptive use (never/ever), breastfeeding (never/ever), HRT use (never/ever) and tubal ligation (no/yes).

^b^Total physical activity was calculated as the sum of moderate and vigorous physical activity.

^c^Overall (ages 12–34) was calculated by summing and averaging the metabolic equivalent of the five predefined age periods.
