# Supplementary material for: Physical Activity During Adolescence and Early-adulthood and Ovarian Cancer Among Women with a BRCA1 or BRCA2 Mutation
Source: Cancer Res Commun. 2023 Nov 28;3(11):2420–9. doi: 10.1158/2767-9764.CRC-23-0223 (PMC10683556; doi:10.1158/2767-9764.CRC-23-0223)
Supplement: Supplementary Table 6 — shows the association between physical activity overall (in MET-hr/week) and ovarian cancer among women with a BRCA1 or BRCA2 mutation. [file crc-23-0223-s06.docx]

**Supplementary Table S6: Association between physical activity overall (in MET-hr/week) and ovarian cancer among women with a *BRCA1* or *BRCA2* mutation.**

| **Physical activity overall**  **(MET-hr/week)^a^** | **Cases/**  **controls** | **Univariate OR (95% CI)** | ***P*** | **Multivariable OR (95% CI)^b^** | ***P*** | **Multivariable OR (95% CI)^c^** | ***P*** |
| --- | --- | --- | --- | --- | --- | --- | --- |
| **Moderate** |  |  |  |  |  |  |  |
| ≤6.75 | 74/74 | Ref. | Ref. | Ref. | Ref. | Ref. | Ref. |
| 6.75 ≤ 21.2 | 80/65 | 1.23 (0.76, 2.00) | 0.41 | 1.32 (0.78, 2.24) | 0.30 | 1.34 (0.79, 2.26) | 0.28 |
| > 21.2 | 51/66 | 0.80 (0.48, 1.31) | 0.37 | 0.77 (0.45, 1.31) | 0.33 | 0.77 (0.45, 1.31) | 0.33 |
| **Vigorous** |  |  |  |  |  |  |  |
| ≤2.8 | 59/70 | Ref. | Ref. | Ref. | Ref. | Ref. | Ref. |
| 2.8 ≤ 27.3 | 81/72 | 1.39 (0.84, 2.31) | 0.20 | 1.34 (0.78, 2.31) | 0.29 | 1.34 (0.78, 2.32) | 0.29 |
| > 27.3 | 65/63 | 1.24 (0.76, 2.01) | 0.39 | 1.17 (0.69, 1.99) | 0.55 | 1.18 (0.70, 2.00) | 0.53 |
| **Total^d^** |  |  |  |  |  |  |  |
| ≤15.4 | 69/69 | Ref. | Ref. | Ref. | Ref. | Ref. | Ref. |
| 15.4 ≤ 47.1 | 70/68 | 1.03 (0.64, 1.68) | 0.90 | 0.99 (0.59, 1.66) | 0.97 | 0.99 (0.59, 1.67) | 0.98 |
| > 47.1 | 66/68 | 0.97 (0.61, 1.55) | 0.91 | 0.94 (0.57, 1.56) | 0.81 | 0.94 (0.56, 1.55) | 0.79 |

Abbreviations: OR, odds ratio; CI, confidence interval.

^a^Overall (ages 12–34) was calculated by summing and averaging the metabolic equivalent of the five predefined age periods.

^b^Adjusted for personal history of breast cancer (no/yes), oral contraceptive use (never/ever), breastfeeding (never/ever), MHT use (never/ever) and tubal ligation (no/yes).

^c^Adjusted for personal history of breast cancer (no/yes), oral contraceptive use (never/ever), breastfeeding (never/ever), MHT use (never/ever), tubal ligation (no/yes) and BMI at age 18.

^d^Total physical activity was calculated as the sum of moderate and vigorous physical activity.
