# Supplementary material for: Physical Activity During Adolescence and Early-adulthood and Ovarian Cancer Among Women with a BRCA1 or BRCA2 Mutation
Source: Cancer Res Commun. 2023 Nov 28;3(11):2420–9. doi: 10.1158/2767-9764.CRC-23-0223 (PMC10683556; doi:10.1158/2767-9764.CRC-23-0223)
Supplement: Supplementary Table 7 — shows the comparison of cases in the matched analysis and larger longitudinal study. [file crc-23-0223-s07.docx]

| **Characteristic** | **Matched cases** | **Cases** | ***P*** |
| --- | --- | --- | --- |
|  | (n = 215) | (n = 2473) |  |
| **Year of baseline questionnaire, mean (range)** | 2010.3 (1996-2018) | 2004.1 (1992-2018) | <0.0001 |
| **Year of birth, mean (range)** | 1957.4 (1935-1985) | 1948.9 (1903-1986) | <0.0001 |
| **Country of residence, n (%)** |  |  | <0.0001 |
| United States | 25 (11.6) | 757 (30.6) |  |
| Canada | 73 (34.0) | 579 (23.4) |  |
| Poland | 113 (52.6) | 665 (26.9) |  |
| Other | 4 (1.8) | 472 (19.1) |  |
| **Mutation, n (%)** |  |  | 0.86 |
| *BRCA1* | 173 (80.5) | 1958 (79.9) |  |
| *BRCA2* | 42 (19.5) | 491 (20.1) |  |
| **Personal history of breast cancer, n (%)** |  |  | 0.07 |
| No | 173 (80.5) | 1849 (74.9) |  |
| Yes | 42 (19.5) | 619 (25.1) |  |

**Supplementary Table S7: Comparison of cases in the matched analysis and larger longitudinal study.**
